# Fish, fans and hydroids: host species of pygmy seahorses

**DOI:** 10.3897/zookeys.103.953

**Published:** 2011-06-10

**Authors:** Bastian T. Reijnen, Sancia E.T. van der Meij, Leen P. van Ofwegen

**Affiliations:** Department of Marine Zoology, Netherlands Centre for Biodiversity Naturalis, Darwinweg 2, 2333 CR Leiden, the Netherlands

**Keywords:** Acanthogorgiidae, Alcyonacea, *Annella*, Anthozoa, *Hippocampus*, host specificity, Hydrozoa, Indo-Pacific, *Muricella*, new associations, Octocorallia, Subergorgiidae

## Abstract

An overview of the octocoral and hydrozoan host species of pygmy seahorses is provided based on literature records and recently collected field data for *Hippocampus bargibanti*, *Hippocampus denise* and *Hippocampus pontohi*. Seven new associations are recognized and an overview of the so far documented host species is given. A detailed re-examination of octocoral type material and a review of the taxonomic history of the alcyonacean genera *Annella* (Subergorgiidae) and *Muricella* (Acanthogorgiidae) are included as baseline for future revisions. The host specificity and colour morphs of pygmy seahorses are discussed, as well as the reliability of (previous) identifications and conservation issues.

## Introduction

Pygmy seahorses (*Hippocampus* spp.) (Pisces: Syngnathidae) are diminutive tropical fish that live in close association with octocorals, colonial hydrozoans, bryozoans, sea grass and algae (Lourie and Kuiter 2008), but little information is available about their host specificity. Most host organisms are notoriously hard to identify because of a lack of clear morphological characters, which leads to the risk of obtaining erroneous identifications. Therefore there is an urgent need for taxonomic revisions of these host species.

The first discovered pygmy seahorse was described as *Hippocampus bargibanti* Whitley, 1970 (redescribed by Gomon 1997) as an associate of the gorgonian *Muricella* sp. In recent years, six other new pygmy seahorse species have been described: *Hippocampus colemani* Kuiter, 2003; *Hippocampus denise* Lourie & Randall, 2003; *Hippocampus pontohi* Lourie & Kuiter, 2008; *Hippocampus satomiae* Lourie & Kuiter, 2008; *Hippocampus severnsi* Lourie & Kuiter, 2008 and *Hippocampus waleananus* Gomon & Kuiter, 2009. An additional species from Japan awaits description (Kuiter 2009). Žalohar et al. (2009) described two fossil seahorse species, one of which (*Hippocampus slovenicus* Žalohar, Hitij & Križnar, 2009) has body ornamentations resembling those of *Hippocampus bargibanti*, *Hippocampus denise*, and *Hippocampus colemani*. It is expected that additional species will be described in the near future (Lourie and Kuiter 2008, Gomon and Kuiter 2009, Kuiter 2009). The diminutive *Hippocampus debelius* Gomon and Kuiter, 2009 belongs to the non-pygmy seahorse species despite its small size and possible association with alcyonarians and/or hydroids. This grouping is based on distinctive characters, i.e. the males’ external tail pouch and separate gill-openings (Gomon and Kuiter 2009, Kuiter 2009), but is not reflected in the classification above species level.

This study deals with the octocoral (Cnidaria: Anthozoa: Octocorallia) and hydrozoan (Cnidaria: Hydrozoa) hosts of the pygmy seahorses *Hippocampus bargibanti*, *Hippocampus denise*, and *Hippocampus pontohi*.The taxonomic problems in the octocoral host genera *Muricella* (Acanthogorgiidae) and *Annella* (Subergorgiidae)are addressed, and type material is re-examined and depicted. In addition, a literature review of all documented host species is provided, as well as accounts on newly recorded associations. The distribution records of pygmy seahorses are updated with four localities in Indonesia and Malaysia.

## Material and methods

**Figure 1.**
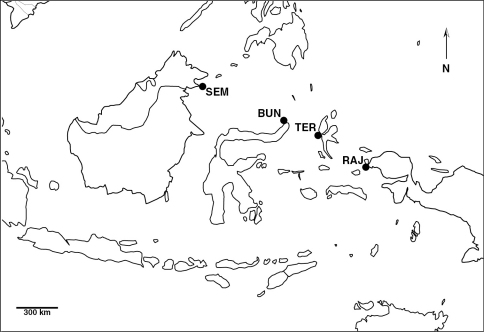
Map showing the fieldwork localities in Indonesia and Malaysia; BUN (Bunaken), RAJ (Raja Ampat), SEM (Semporna) and TER (Ternate).

The majority of the pygmy seahorse records in the present study was obtained during fieldwork in Raja Ampat, West Papua, Indonesia (2007). Additional observations were made in Bunaken National Marine Park, North Sulawesi (2008), Ternate and Halmahera, North Moluccas (2009) in Indonesia, and Semporna, eastern Sabah, in Malaysia (2010) (Fig. 1).

**Figure 2.**
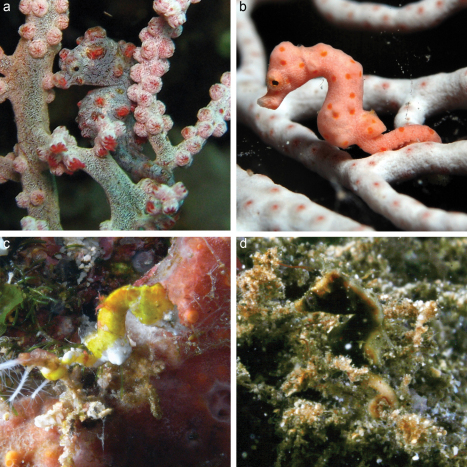
*In-situ* photographs **A** *Hippocampus bargibanti* on *Muricella* sp. 3 (RMNH Coel. 39866, see Fig. 7), Turtles Reef, Raja Ampat (photo F.R. Stokvis) **B** *Hippocampus denise* on *Annella reticulata* (RMNH Coel. 39880, see Fig. 10); W Mansuar, Raja Ampat (photo B.W. Hoeksema) **C** *Hippocampus pontohi* (host not collected) Timur I, Bunaken (photo S.E.T. van der Meij) **D** *Hippocampus severnsi* (host not collected) Siladen I, SE Siladen (photo B.T. Reijnen).

Soft corals, gorgonians and hydrozoans were thoroughly searched for pygmy seahorses to a maximum depth of 40 m (using SCUBA), with the help of local dive guides where available (Raja Ampat, Bunaken). *In situ* photographs were taken of both the hosts and the associated seahorses (Fig. 2). The total number of seahorses per host colony was counted, the height of each host colony was estimated and a sample was taken for identification and as voucher material. All material is stored on 70% ethanol in the collections of NCB Naturalis, Leiden (catalogue numbers RMNH Coel.). Subsamples of the Ternate material are deposited in the collections of Museum Zoologicum Bogoriense (Java, Indonesia).

For the identification of the octocoral hosts, microscope slides and SEM photographs of the sclerites were made. These were obtained by dissolving the octocoral tissue in 10% sodium hypochlorite, after which they were rinsed five times with tap water and five times with double-distilled water. The sclerites were subsequently dried on glass microscope slides on a hot plate. After drying, the sclerites were brushed on a SEM stub and coated with platinum. A JEOL JSM6480LV electron microscope operated at 10 kV was used for the SEM photography. The hydrozoans were identified using a dissecting microscope.

## Results

In the literature eight pygmy seahorse species have been recorded as associates of hydroids and octocorals (Table 1). During the fieldwork a total of 10 observations of *Hippocampus bargibanti*,10 of *Hippocampus denise*,four of *Hippocampus pontohi* and one of *Hippocampus severnsi* was made. The total number of encountered pygmy seahorse individuals is 52 (Table 2), in which 10 host species were involved. For *Hippocampus bargibanti* and *Hippocampus denise* there is overlap in host species with the previous records. The present host records of *Hippocampus pontohi* do not correspond with previous ones (Table 1). On one occasion *Hippocampus severnsi* was observed, but the host organism was not sampled.

**Table 1.** Distribution ranges of pygmy seahorses and their host species associations as obtained from literature.

Species	Confirmed distribution	Host species	Reference
*Hippocampus bargibanti*	Australia, New Caledonia, Indonesia, Japan, Papua New Guinea, Philippines	*Muricella paraplectana* Grasshoff, 1999	Whitley 1970, Gomon 1997, Grasshoff 1999, Lourie 2001, Lourie and Randall 2003, Fricke 2004, Lourie et al. 2004, Baine et al. 2008, Lourie and Kuiter 2008
		*Muricella plectana* Grasshoff, 1999	
		*Muricella* sp.	
*Hippocampus colemani*	Australia (Lord Howe Isl.)	*Halophila* sp.	Kuiter 2003
		*Zostera* sp.	
*Hippocampus denise*	Indonesia, Malaysia, Micronesia, Palau, Papua New Guinea, Philippines, Solomon Isl., Vanuatu	*Annella mollis* (Nutting, 1910)	Lourie 2001, Lourie and Randall 2003, Lourie et al. 2004, Lourie and Kuiter 2008, Smith and Tibbetts 2008
		*Annella reticulata* (Ellis & Solander, 1786)	
		*Muricella* sp.	
		?*Acanthogorgia* spp.	
		?*Echinogorgia* sp.	
		?*Subergorgia* sp.	
*Hippocampus pontohi*	Indonesia (widespread)	*Aglaophenia cupressina* Lamouroux, 1812	Lourie and Kuiter 2008, Kuiter 2009
		*Halimeda* sp.	
*Hippocampus satomiae*	Indonesia (E Kalimantan, N Sulawesi), Malaysia (N Borneo)	?*Carijoa* sp.	Lourie and Kuiter 2008, Kuiter 2009
		*Nephthea* sp.	
*Hippocampus severnsi*	Indonesia, Japan, Papua New Guinea, Solomon Isl., Fiji	*Antennellopsis integerrima* Jäderholm, 1919	Kuiter 2000, Lourie and Kuiter 2008
		*Catenicella* sp.	
		*Halicordyle disticha* [*Halocordyle disticha* (Goldfuss, 1820) = *Pennaria disticha* (Goldfuss, 1820)]	
		*Halimeda* sp.	
		*Lytocarpus phoeniceus* (Busk, 1852)	
		*Muricella* sp.	
		?*Menella* sp.	
*Hippocampus waleananus*	Indonesia (Walea Isl., Togian Isl.)	*Nephthea* sp.	Gomon and Kuiter 2009
*Hippocampus* sp. A	Japan (Hachijo Isl., Izu Isl.)	unknown	Kuiter 2009

**Table 2.** Host-species associations as recorded in this study, no. = the number of observed pygmy seahorses per colony. Observations recorded in Raja Ampat, unless otherwise stated.

Species	No.	Host species	RMNH Coel.	Colony height	Depth	Locality	Lat.	Long.
*Hippocampus bargibanti*	1	*Muricella* sp. 1	39868	30 cm	16 m	S Friwin Isl.	0°28’54.54”S; 130°41’54.06”E
	2	*Muricella* sp. 1	39871	100 cm	21 m	Mike’s Point, SE Gam Kerupiar Isl.		0°30’57.06”S; 130°40’22.14”E
	6	*Muricella* sp. 1	39874	100 cm	22 m	Maitara NW (Ternate)	0°44’19.21”N; 127°20’59.99”E	
	1	*Muricella* sp. 2	39869	70 cm	21 m	S Friwin Isl.		0°28’54.54”S; 130°41’54.06”E
	1	*Muricella* sp. 3	39864	30 cm	18 m	Mike’s Point, SE Gam Kerupiar Isl.	0°30’57.06”S; 130°40’22.14”E	
	4	*Muricella* sp. 3	39865	60 cm	14 m	Sorido wall, E Kri		0°33’13.20”S; 130°41’16.91”E
	1	*Muricella* sp. 3	39866	70 cm	12 m	Turtles Reef	0°32’35.16”S; 130°41’51.06”E	
	1	*Muricella* sp. 3	39867	25 cm	18 m	Mike’s Point, SE Gam Kerupiar Isl.		0°30’57.06”S; 130°40’22.14”E
	5	*Muricella* sp. 3	39870	70 cm	23 m	Mike’s Point, SE Gam Kerupiar Isl.	0°30’57.06”S; 130°40’22.14”E	
	1	*Muricella* sp. 3	39872	80 cm	23 m	NW Batanta		0°47’45.78”S; 130°30’21.24”E
*Hippocampus denise*	1	*Annella mollis*	39875	50 cm	20 m	Mike’s Point, SE Gam Kerupiar Isl.	0°30’57.06”S; 130°40’22.14”E	
	1	*Annella mollis*	39876	80 cm	20 m	Sleeping barracuda		0°32’43.14”S; 130°42’01.62”E
	1	*Annella cf. mollis*	39877	100 cm	22 m	S Kri, Kri Isl.	0°33’32.26”S; 130°41’15.48”E	
	1	*Annella cf. mollis*	39881	50 cm	22 m	Mike’s Point, SE Gam Kerupiar Isl.		0°30’57.06”S; 130°40’22.14”E
	4	*Annella reticulata*	39878	60 cm	24 m	W Mansuar	0°30’41.76”S; 130°33’35.34”E	
	3	*Annella reticulata*	39879	40 cm	22 m	W Mansuar		0°30’41.76”S; 130°33’35.34”E
	4	*Annella reticulata*	39880	30 cm	24 m	W Mansuar	0°30’41.76”S; 130°33’35.34”E	
	4	*Annella reticulata*	39882	30 cm	22 m	Yeffam Isl., NW Pulau Keruo		0°35’15.36”S; 130°17’42.66”E
	1	*Annella reticulata*	39952	60 cm	22 m	Timba Timba Isl. (Semporna)	4°33’37.70”N; 118°55’30.40”E	
	1	*Muricella* sp. 2	39873	100 cm	20-25 m	Yeffam Isl., NW Pulau Keruo		0°35’15.36”S; 130°17’42.66”E
*Hippocampus pontohi*	2	not collected	–	–	–	Timur I (Bunaken)	1°36’38.46”N; 124°46’58.74”E	
	1	*Thyroscyphus fruticosus* (Esper, 1793)	39883	20 cm	8 m	Nikson, SE Mansuar		0°34’51.42”S; 130°38’31.62”E
	1	*Thyroscyphus fruticosus* (Esper, 1793) / *Lytocarpia phyteuma* (Kirchenpauer, 1876)	39884 / 39886	–	26 m	S Kri, Kri Isl.	0°33’32.26”S; 130°41’15.48”E	
	1	*Clytia cf. gravieri* (Billard, 1904)	39885	–	20 m	Mioskon Isl.		0°29’48.48”S; 130°43’37.38”E
*Hippocampus severnsi*	3	not collected	–	–	–	Siladen I, SE Siladen (Bunaken)	1°37’30.66”N; 124°47’53.88”E	

### Anthozoa: Alcyonacea

**Family Acanthogorgiidae Gray, 1859**

**Genus *Muricella* Verrill, 1869**

*Muricella* Verrill, 1869: p. 450

*Muricella* Bayer, 1981: p. 920, 945

*Muricella* Grasshoff, 1999: p. 33

**Figure 3.**
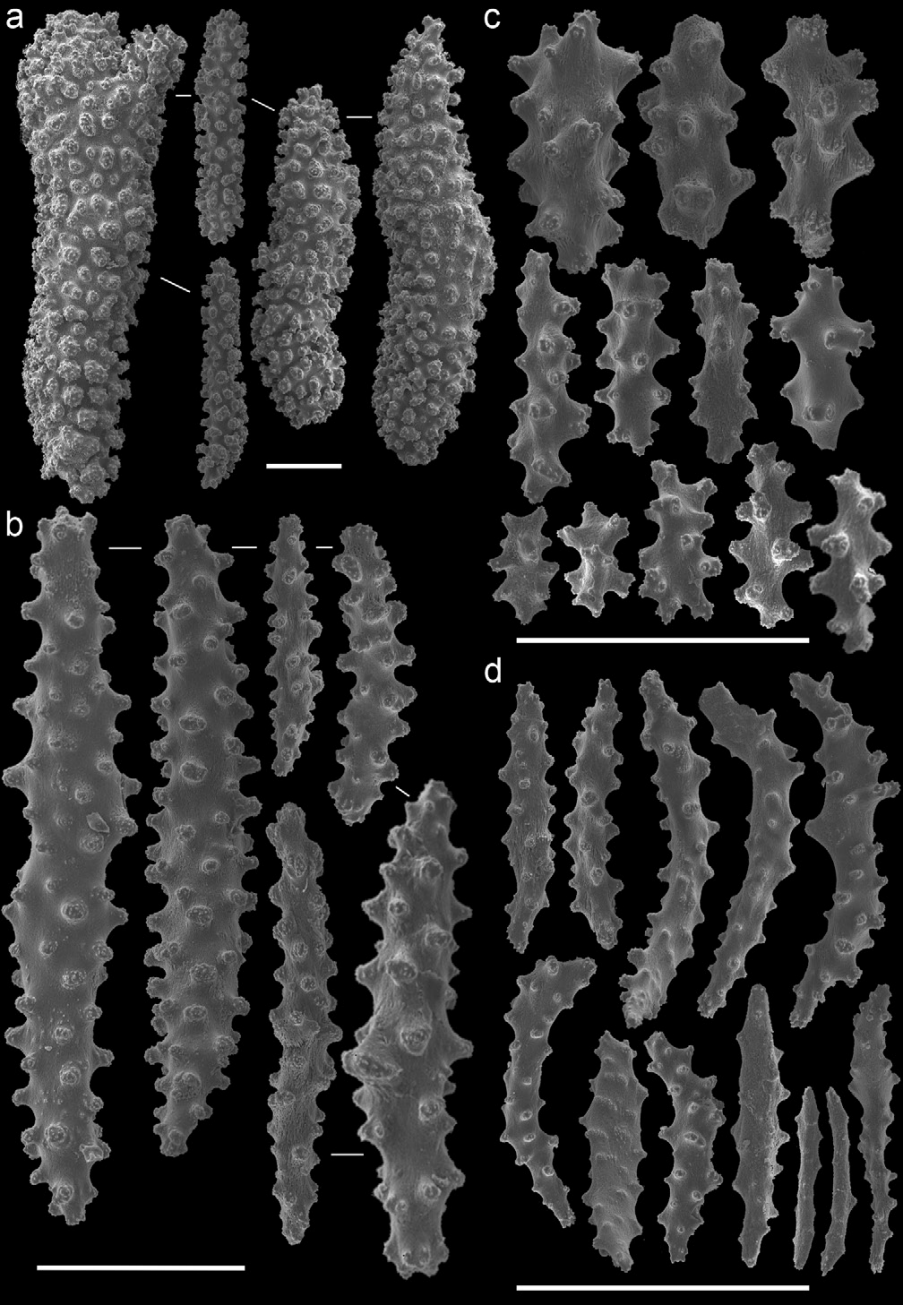
*Muricella plectana* (MNHN, HG-100 - holotype)**A** spindles from coenenchyme and polyp **B** smaller spindles from coenenchyme and polyp **C** capstans from adaxial layer **D** rods from tentacle. Scale bars represent 0.1 mm.

**Figure 4.**
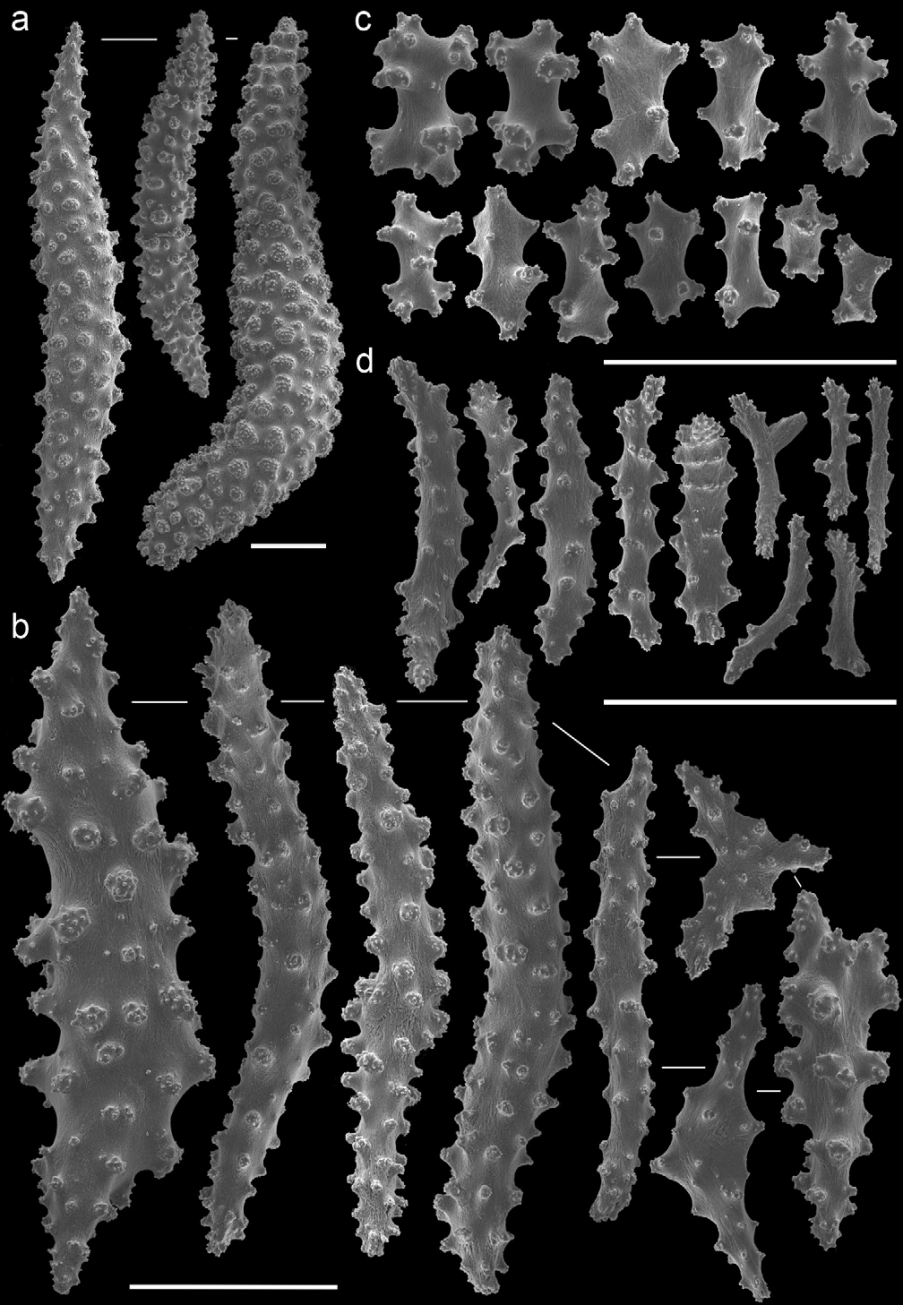
*Muricella paraplectana* (MNHN, HG-121 - holotype)**A** spindles from coenenchyme and polyp **B** smaller spindles from coenenchyme and polyp **C** capstans from adaxial layer **D** rods from tentacle. Scale bars represent 0.1 mm.

**Figure 5.**
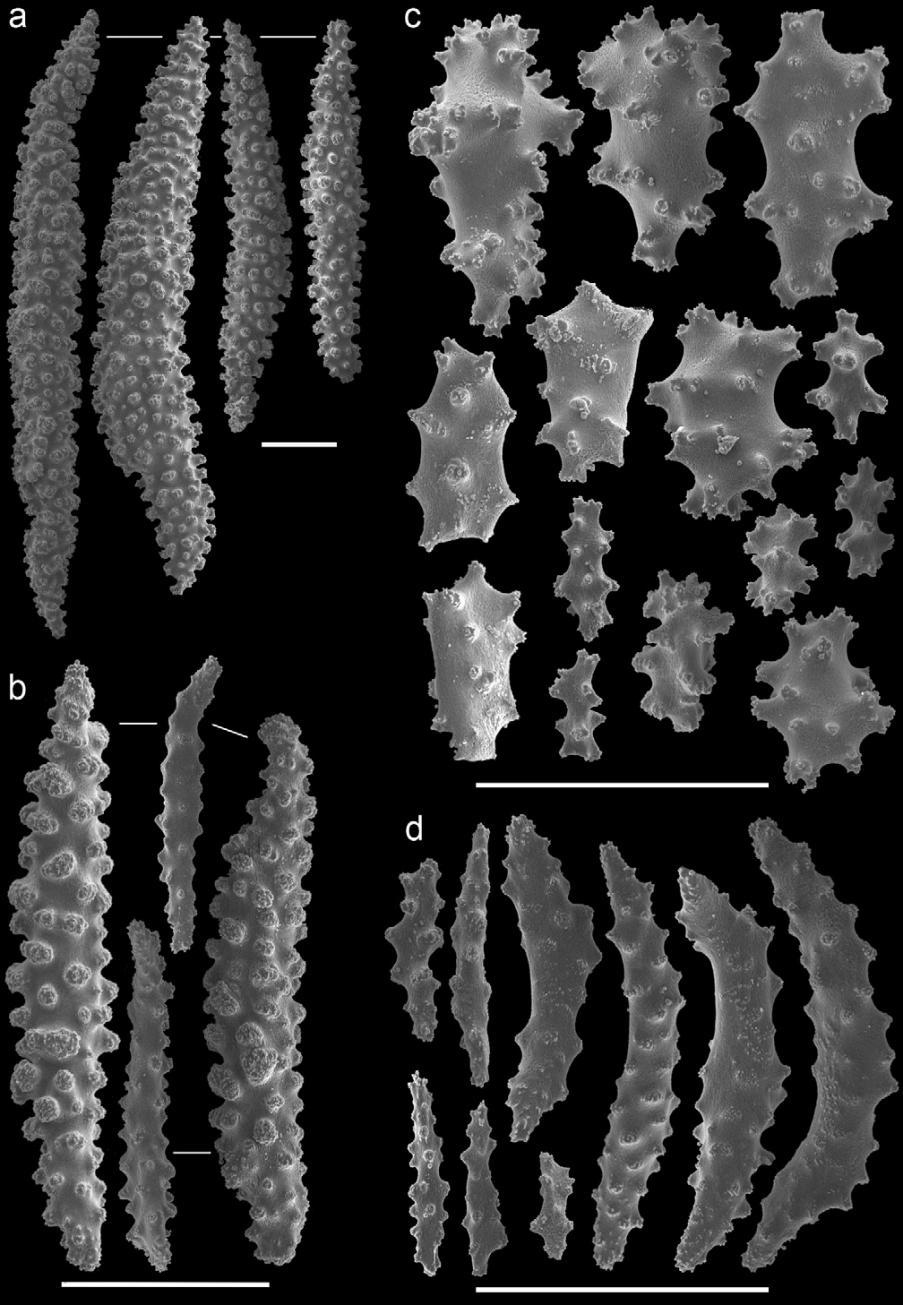
*Muricella* sp. 1 (RMNH Coel. 39871)**A** spindles from coenenchyme and polyp **B** smaller spindles from coenenchyme and polyp **C** capstans from adaxial layer **D** rods from tentacle. Scale bars represent 0.1 mm.

**Figure 6.**
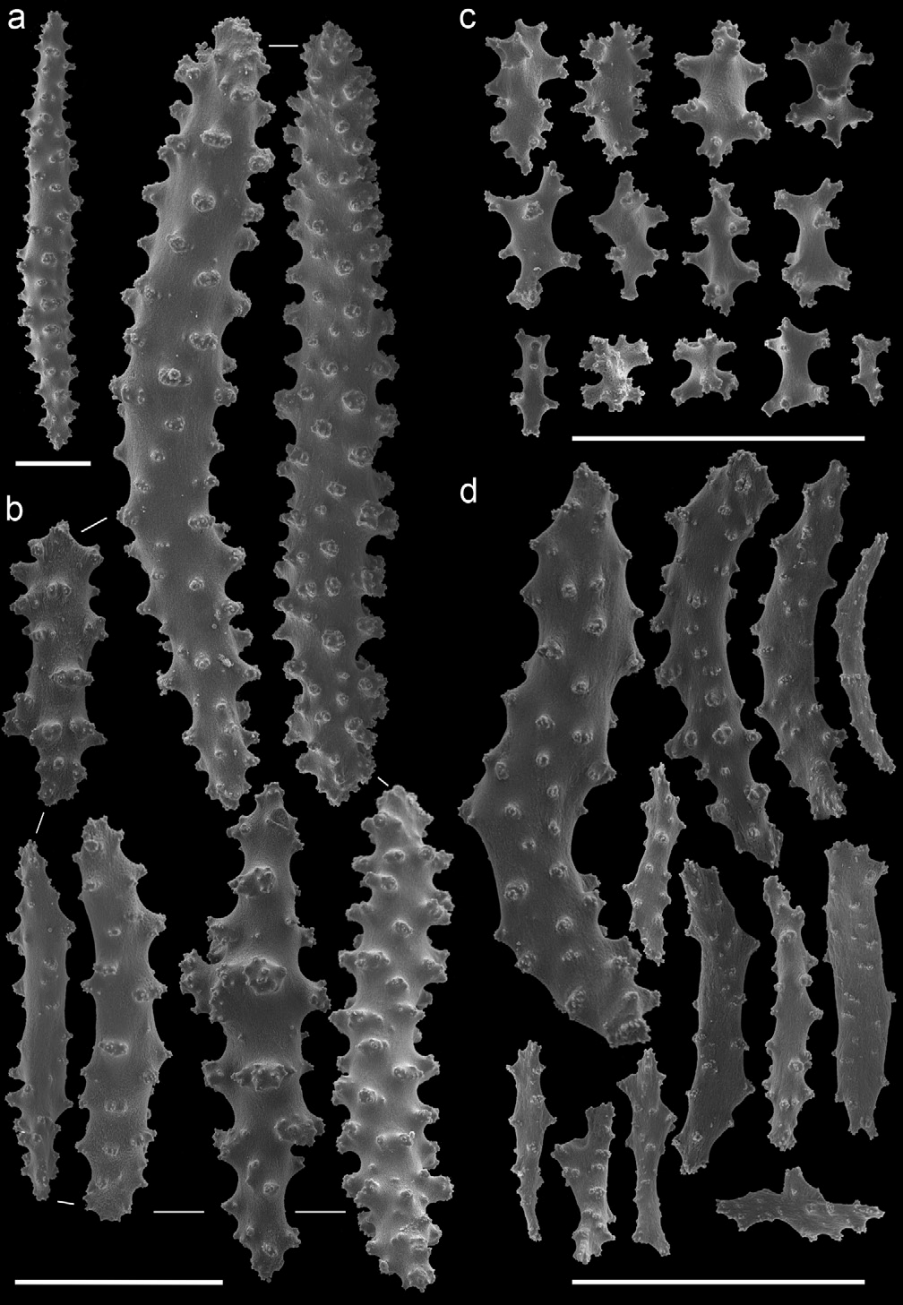
*Muricella* sp. 2 (RMNH Coel. 39873) **A** spindle from coenenchyme **B** smaller spindles from coenenchyme and polyp **C** capstans from adaxial layer **D** rods from tentacle. Scale bars represent 0.1 mm.

**Figure 7.**
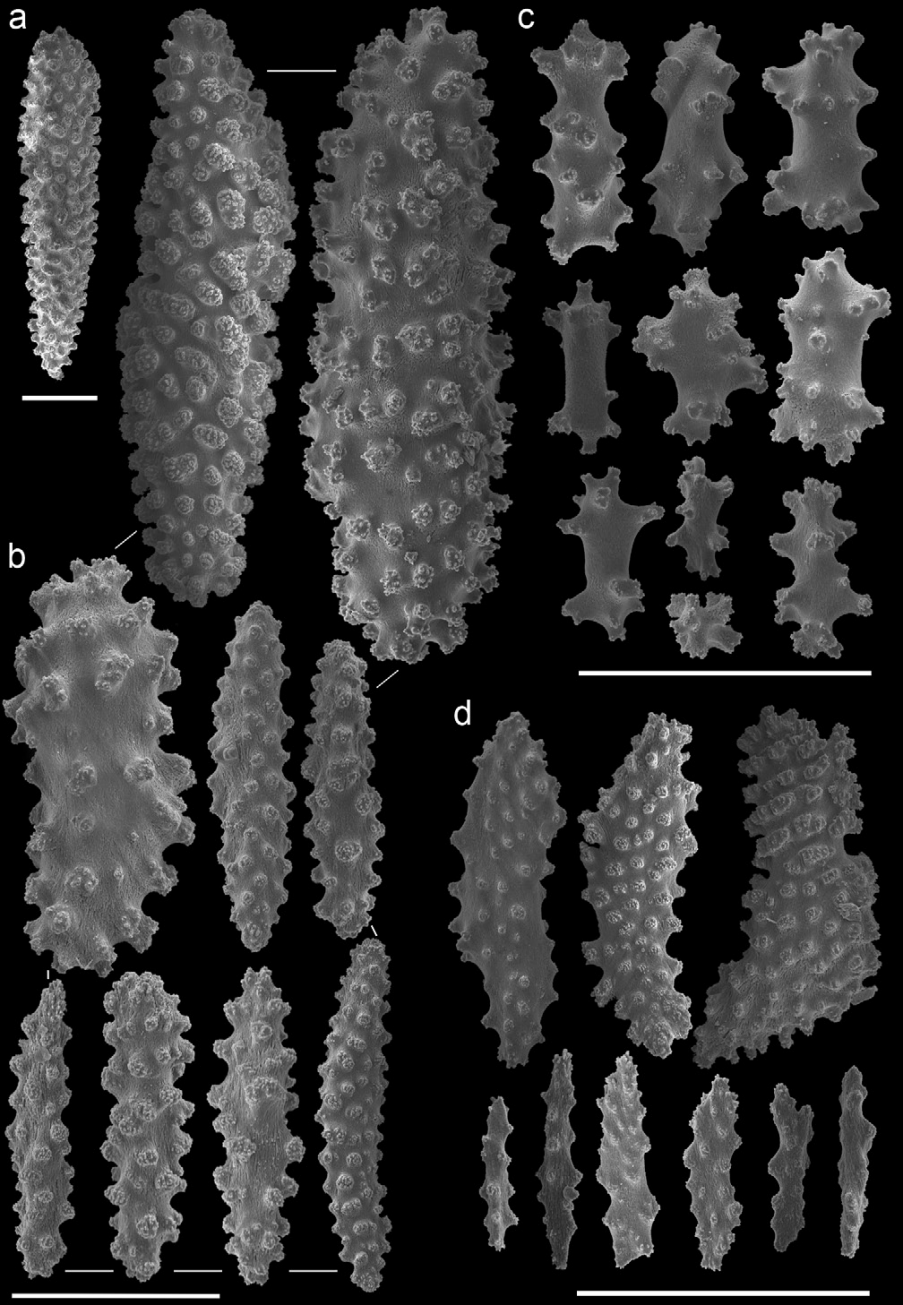
*Muricella* sp. 3 (RMNH Coel. 39865) **A** spindle from coenenchyme **B** smaller spindles from coenenchyme and polyp **C** capstans from adaxial layer **D** rods from tentacle. Scale bars represent 0.1 mm.

In the remarks of the species descriptions of *Muricella plectana* Grasshoff, 1999, and *Muricella paraplectana* Grasshoff, 1999, from New Caledonia, Grasshoff (1999) already mentioned that both species are hosts to *Hippocampus bargibanti*. Since the species descriptions of *Muricella plectana* and *Muricella paraplectana* are not in accordance with the depicted sclerites (Grasshoff, 1999; Figs 50–51), the holotypes have been re-examined by microscope slides and SEM photography (Figs 3–4). The material included in the present study was compared with these holotypes, but clearly does not belong to these two species. Consequently, three additional host species can be recognized for *Hippocampus bargibanti*. A total of 35 nominal species are currently accepted within the genus *Muricella* (Ofwegen 2010). Since a revision of this genus is lacking, the species names cannot be given. Therefore the sclerite morphology of *Muricella plectana*, *Muricella paraplectana* and *Muricella* sp. 1–3 is provided (figs 3–7).

Firstly, *Muricella* sp. 1 (RMNH Coel. 39868, 39871, 39874) is characterized by wide, plump, capstans from the adaxial layer, up to 0.12 mm long (Fig. 5). Secondly, *Muricella* sp. 2 (RMNH Coel. 39869, 39873) is characterized by small, slender adaxial capstans, up to 0.05 mm long (Fig. 6). Thirdly, *Muricella* sp. 3 (RMNH Coel. 39864-67, 39870, 39872), is characterized by adaxial capstans intermediate in shape between the first two, up to 0.10 mm long, and big spindles with rounded ends (Fig. 7). The latter are lacking in the first two species. *Muricella plectana* has similar plump spindles in the coenenchyme but differs from the present material by lacking the bent spindles from the polyp (Fig. 3). *Muricella paraplectana* differs from all other material by having spindles with pointed ends (Fig. 4).

**Family Subergorgiidae Gray, 1859**

**Genus *Annella* Gray, 1858**

*Annella* Gray, 1858: p. 287

*Suberogorgia* Stiasny, 1937: p. 83

*Subergorgia* Bayer, 1981: p. 910

*Annella* Grasshoff, 1999: p. 16

According to Grasshoff (1999), *Annella* comprises reef-dwelling Indo-Pacific subergorgiids that form netlike fans. Two species are currently recognized, *Annella mollis* (Nutting, 1910) and *Annella reticulata* (Ellis and Solander, 1786). These species can be distinguished by their growth form: *Annella mollis* by elongated meshes in the central part of the fan and *Annella reticulata* by having small polygonal meshes.

**Figure 8.**
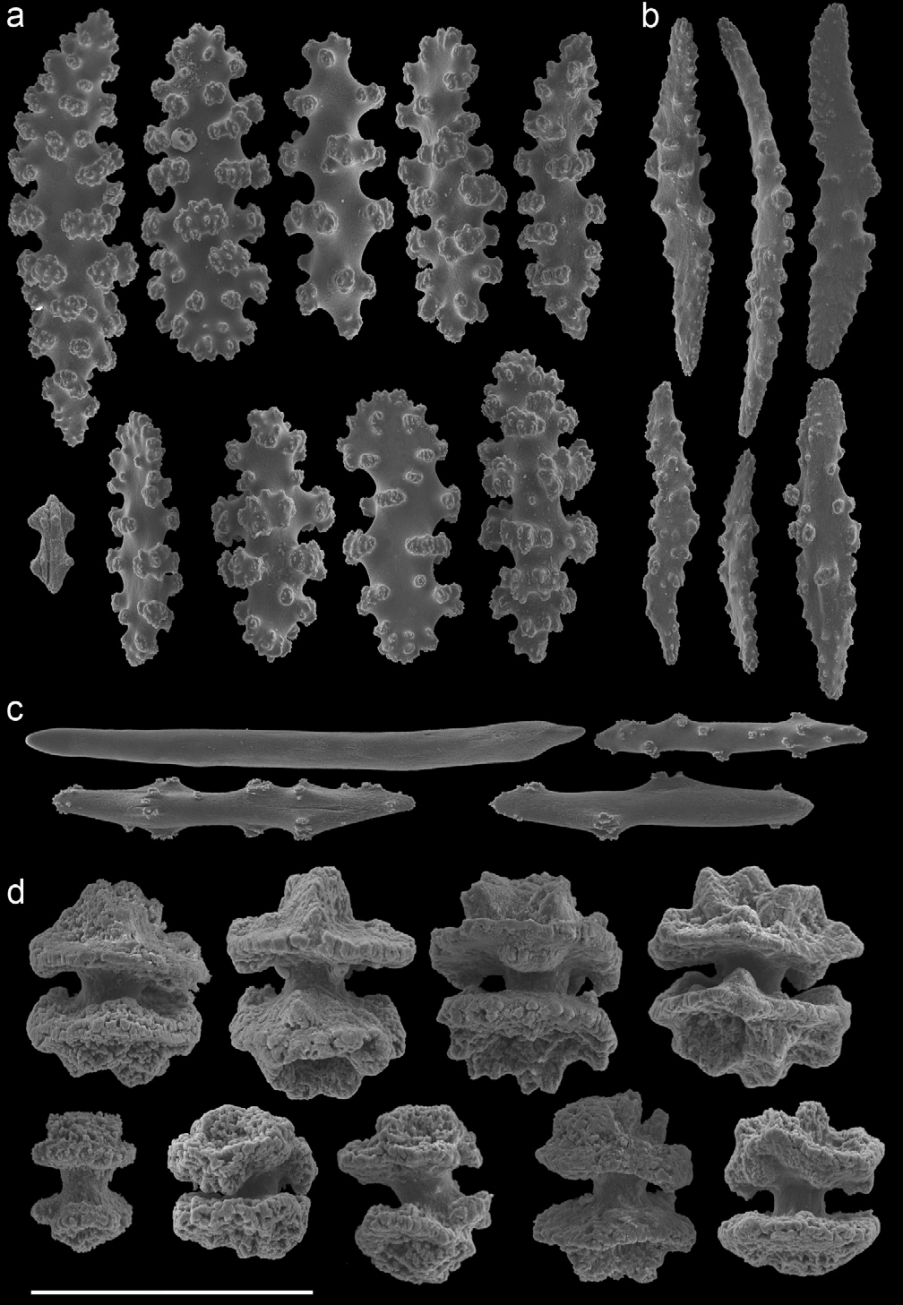
*Euplexaura reticulata* (ZMA Coel. 3504 - holotype) **A** spindles from the coenenchyme **B** tentacle rods **C** medulla spindles from the axis **D** double heads from the surface layer. Scale bar represents 0.1 mm, except for **D** which is 0.05 mm.

The taxonomic history of the genus *Annella* is puzzling. Ellis and Solander (1786) described *Gorgonia reticulata* and added a drawing of the habitus without further description or indication of its type locality. The type specimen of *Gorgonia reticulata* is presumably lost. Subsequently, Gray (1857[1858]) described the genus *Annella*, with *Annella reticulata* as type species, but it is unknown whether he associated this species with *Gorgonia reticulata*.Later, Nutting (1910) described *Euplexaura reticulata* (Fig. 8), probably without considering a possible homonymy involving *Annella reticulata* and *Gorgonia reticulata*. Stiasny (1937) synonymised *Gorgonia reticulata* and *Euplexaura reticulata*,based on the external morphology, and transferred the species to *Suberogorgia reticulata*.Grasshoff (1999) eventually placed *Gorgonia reticulata*, *Annella reticulata*, *Euplexaura reticulata* and *Suberogorgia reticulata* in the genus *Annella*. The species is therefore currently known as *Annella reticulata* (Ellis and Solander, 1786). Here the holotype of *Euplexaura reticulata* is re-examined and considered different from *Annella reticulata*, based on the morphology of the double head sclerites (Figs 8, 10). Due to the netlike structure of these gorgonians, it is not surprising that the different authors independently chose ‘reticulata’ as epithet, so adding to the confusion.

Nutting (1910) described a different species as *Euplexaura mollis* (type locality Moluccas). Stiasny (1937) transferred this species to *Suberogorgia* [= *Subergorgia*] (Bayer, 1981), and subsequently Grasshoff (1999) placed itin the genus *Annella*.The species is therefore currently known as *Annella mollis* (Nutting, 1910).

A taxonomic revision of *Annella* has not yet been made, but Grasshoff (2001) provided an overview of the sclerite diversity observed within this genus. He suggests that the morphological diversity of the sclerites within these two species is correlated with their geographical distribution in the Indo-Pacific. To the best of our knowledge this would be the first and only case in octocoral taxonomy, in which sclerite morphology varies geographically. Following Grasshoff’s (2001) overview of the sclerites, the *Annella* specimens were compared with the nearest region used by Grasshoff (2001), *viz*. the Moluccas. Based on those characters five specimens are identified as *Annella reticulata* (RMNH Coel. 39878-80, 39882, 39952; Fig. 10).Likewise, two specimens are identified as *Annella mollis* (RMNH Coel. 39875-76; Fig. 11), although the double heads of the examined specimens are less developed compared to Grasshoff’s *Annella mollis* from the Moluccas. Two of the specimens with an *Annella mollis* colony form had sclerites like the ones depicted for specimens from the Maldives (Grasshoff 2001). These two specimens are provisionally identified as *Annella* cf. *mollis* (RMNH Coel. 39877, 39881; Fig. 12) and share similarities with the holotype of *Euplexaura reticulata* (Fig. 8).

**Figure 9.**
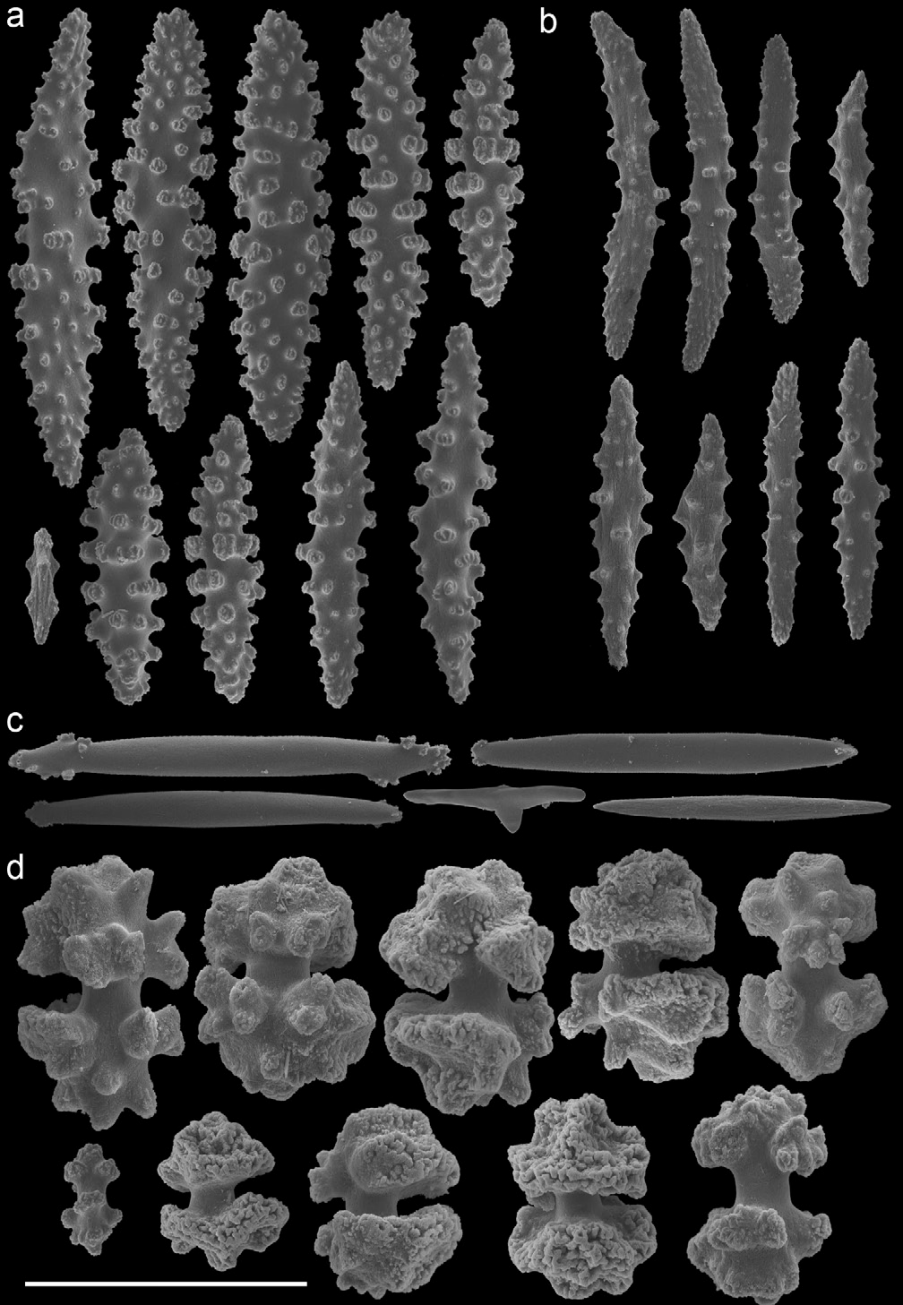
*Euplexaura mollis* (ZMA Coel. 3498 - holotype) **A** spindles from the coenenchyme **B** tentacle rods **C** medulla spindles from the axis **D** double heads from the surface layer. Scale bar represents 0.1 mm, except for **D** which is 0.05 mm.

The sclerites of the holotype of *Euplexaura mollis* from the Moluccas (= *Annella mollis* sensu Grasshoff 1999, 2000) (Fig. 9) were also examined and compared with those pictured by Grasshoff (2001). These sclerites resemble the sclerites in drawings of *Annella reticulata* from the Moluccas instead of those of *Annella mollis*, whereas the habitus resembles *Annella mollis*. Based on our presented material and additional material from the NCB Naturalis collection it seems unlikely that the sclerites of the two *Annella* species differ according to locality. Most varieties, as described by Grasshoff concerning the geographic areas, are also found in Indonesian and Malaysia’s seas (unpublished data). Additional material from other locations is needed to test Grasshoff’s hypothesis on geographically determined sclerite morphotypes.

### Hydrozoa

On four occasions specimens of *Hippocampus pontohi* were observed and three of their hosts were collected. Two records of *Hippocampus pontohi* individuals are from a colony of *Thyroscyphus fruticosus* (Esper, 1793) (RMNH Coel. 39883-4), a common littoral species on coral reefs with a distribution range throughout Indonesia (Prof. W. Vervoort, pers. comm.). A single individual from Kri Island (Raja Ampat) was found on a specimen of *Thyroscyphus fruticosus* intertwined with a specimen of the hydroid *Lytocarpia phyteuma* (Kirchenpauer, 1876) (RMNH Coel. 39886), therefore both co-host species are listed in Table 2. *Lytocarpia phyteuma* is an uncommon hydrozoan, which can be found at 0–50 m depth, especially in eastern Indonesia (Prof. W. Vervoort, pers. comm.). The *Hippocampus pontohi* individualfrom Mioskon Island was found on specimens of *Clytia* cf. *gravieri* (Billard, 1904) (RMNH Coel. 39885), a common hydrozoan on coral reefs with a wide (sub-) tropical distribution range. Due to the small amount of collected material, a positive identification is not possible. This hydroid species was also recorded during previous expeditions in Indonesia, such as the Snellius II expedition (1983–84) (unpublished data Prof. W. Vervoort). The host of *Hippocampus severnsi* was unfortunately not sampled and therefore its identity remains unknown.

## Discussion

Many sessile marine organisms contribute to the high marine biodiversity in the so-called Coral Triangle by acting as host for many associated organisms (Hoeksema 2007). Gorgonians are hosts to a variety of species, such as sponges, molluscs, hydroids, crustaceans, brittle stars and fish (Munday et al. 1997, Goh et al. 1999, McLean and Yoshioka 2007, Neves et al. 2007, Puce et al. 2008, Sih and Chouw 2009, Reijnen et al. 2010). The ‘persistence’ of the relationship (intermittent occurrence on host) between the associated fauna and the host organism is often largely unknown (Goh et al.1999).

Pygmy seahorses were observed to remain on a single gorgonian for periods of at least 3–40 weeks. Information on the pygmy seahorse whereabouts after this period is lacking, and movement between different hosts was not directly observed (Baine et al. 2008). The claim that pygmy seahorses appear to parasitize their hosts (Kuiter 2000, Teske et al. 2004) has not been substantiated, just like the observations that species were seen moving over a mushroom coral (*Fungia* sp.) and encrusting sponges (Lourie and Kuiter 2008) do not seem to be related to real host specificity.

### Host specificity

In the case of *Hippocampus bargibanti* two host species have been recorded in the literature, *Muricella plectana* and *Muricella paraplectana*.Three additional *Muricella* species from the Indo-Pacific, different from *Muricella plectana* or *Muricella paraplectana*,were found during the present study. *Hippocampus bargibanti* is therefore associated with at least five different *Muricella* spp.Unfortunately, the genus *Muricella* is in need of a revision (Samimi and Ofwegen 2009). The latest overview of the genus *Muricella* was made by Kükenthal (1924), in which species-specific characters are usually missing. This makes it impossible to identify specimens to species level. Since Kükenthals’ overview only three additional *Muricella* species have been described (Grasshoff 1999, 2000), which are considered endemic to New Caledonia and the Red Sea. Although the current status of the taxonomy of this gorgonian genus is a large obstacle in identifying species, the results herein indicate that *Muricella* sp. 1, *Muricella* sp. 2 and *Muricella* sp. 3 are new host records for *Hippocampus bargibanti*, each based on their own unique characters (Figs 5–7).

Individuals of *Hippocampus denise* primarily occur on colonies of *Annella* spp., which they strongly resemble in colour pattern, resulting in an optimal camouflage. Based on our results *Hippocampus denise* lives in association with at least three different *Annella* species: *Annella reticulata*, *Annella mollis* and *Annella* cf. *mollis*. No other *Annella* species are currently recognized, and a revision of the genus *Annella* is needed. This will most likely show that additional *Annella* species await description. One individualof *Hippocampus denise* was found on *Muricella* sp. 2 (Fig. 13). This association was already known (Table 1), but appears quite unusual. Additional host genera for *Hippocampus denise* are expected, based on published photographs (Kuiter 2009). A study by Sih and Chouw (2009) showed that other fish species (*Bryaninops amplus* Larson, 1985) associated with gorgonian hosts, select their habitat on physical properties, such as the host’s size and surface area, rather than the species to which it belongs. This may explain why *Hippocampus denise* was encountered on a *Muricella* sp., instead of on its far more common host genus *Annella*. Fish species may generally be more associated with certain host gorgonians, but they can still be found on other hosts if the preferred host is not available.

### Colour morphs

Different colour morphs are recorded for the gorgonian-associated species *Hippocampus bargibanti*, and *Hippocampus denise* (Lourie et al.2004, Kuiter 2009), resulting in the most optimal camouflage considering the colour and the polyp structure of the gorgonians, which are perfectly mimicked by the pygmy seahorses. According to Lourie et al.(2004) the pale grey, purple with pink, and red tubercles colour morphs of *Hippocampus bargibanti* are found on *Muricella plectana*,whereas species showing yellow with orange tubercles are found on *Muricella paraplectana*. Unfortunately, it remains uncertain whether this is a valid assumption, without examining the host’s sclerites. Neither *Muricella plectana* nor *Muricella paraplectana* were encountered during our field studies and all our specimens of *Hippocampus bargibanti* belonged to the pale grey / purple colour morpHippocampus The strict association between colour morph and specific host species can therefore not be confirmed. Based on the present data such strict associations seem unlikely, since identically coloured host species are in fact often different species.

For other pygmy seahorse species new colour morphs may be encountered, since pygmy seahorses are enigmatic species that are popular objects for divers and underwater photographers. As a result they often appear in dive magazines and field guides. Occasionally these pictures show new colour morphs or maybe even new species. For future research such observations and sightings can contribute to the general knowledge and ecology of pygmy seahorses, especially when the host organisms are collected for taxonomic studies.

### Reliability of identifications

**Figure 10.**
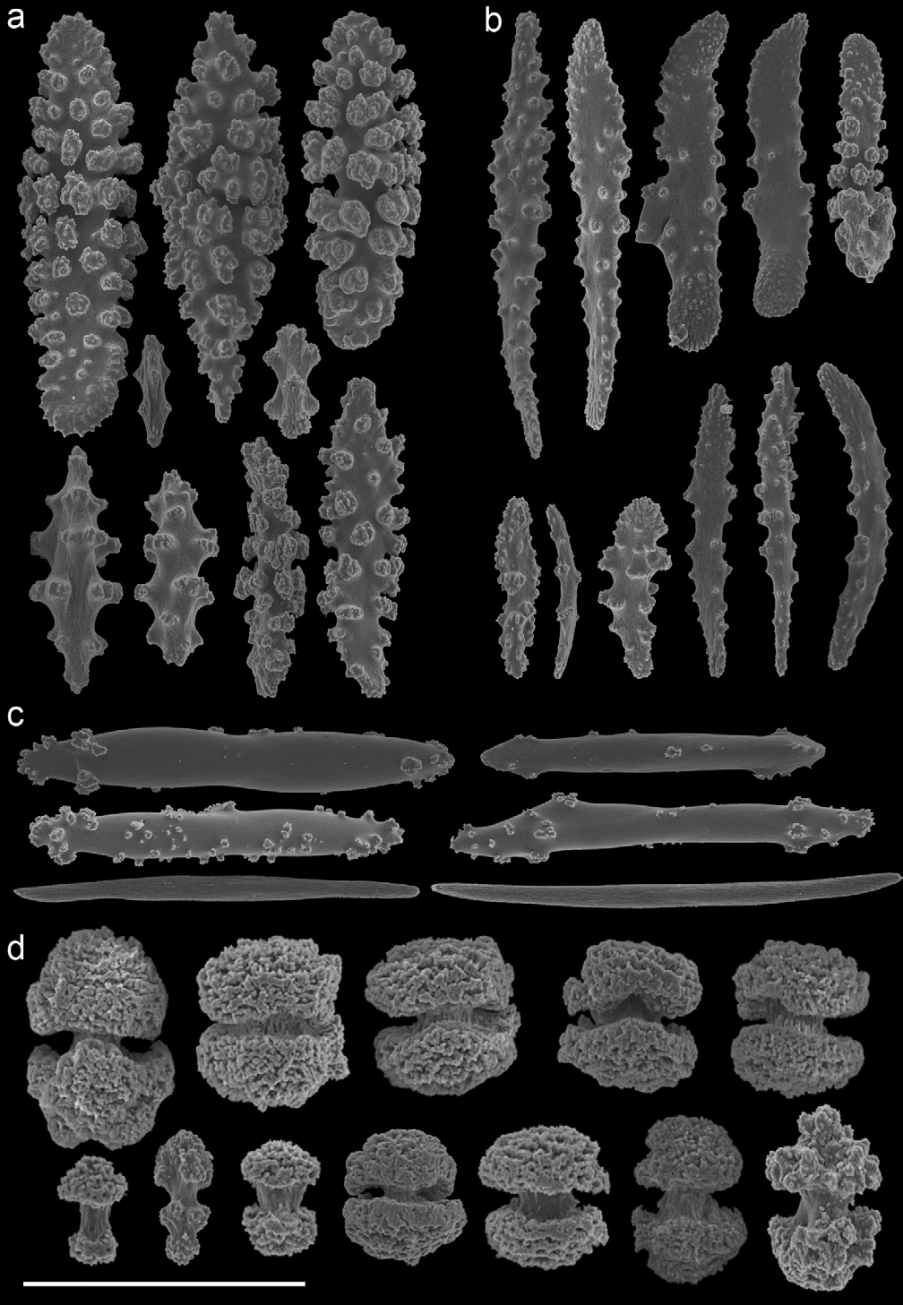
*Annella reticulata* (RMNH Coel. 39882) **A** spindles from the coenenchyme **B** tentacle rods **C** medulla spindles from the axis (d) double heads from the surface layer. Scale bar represents 0.1 mm, except for **D** which is 0.05 mm.

**Figure 11.**
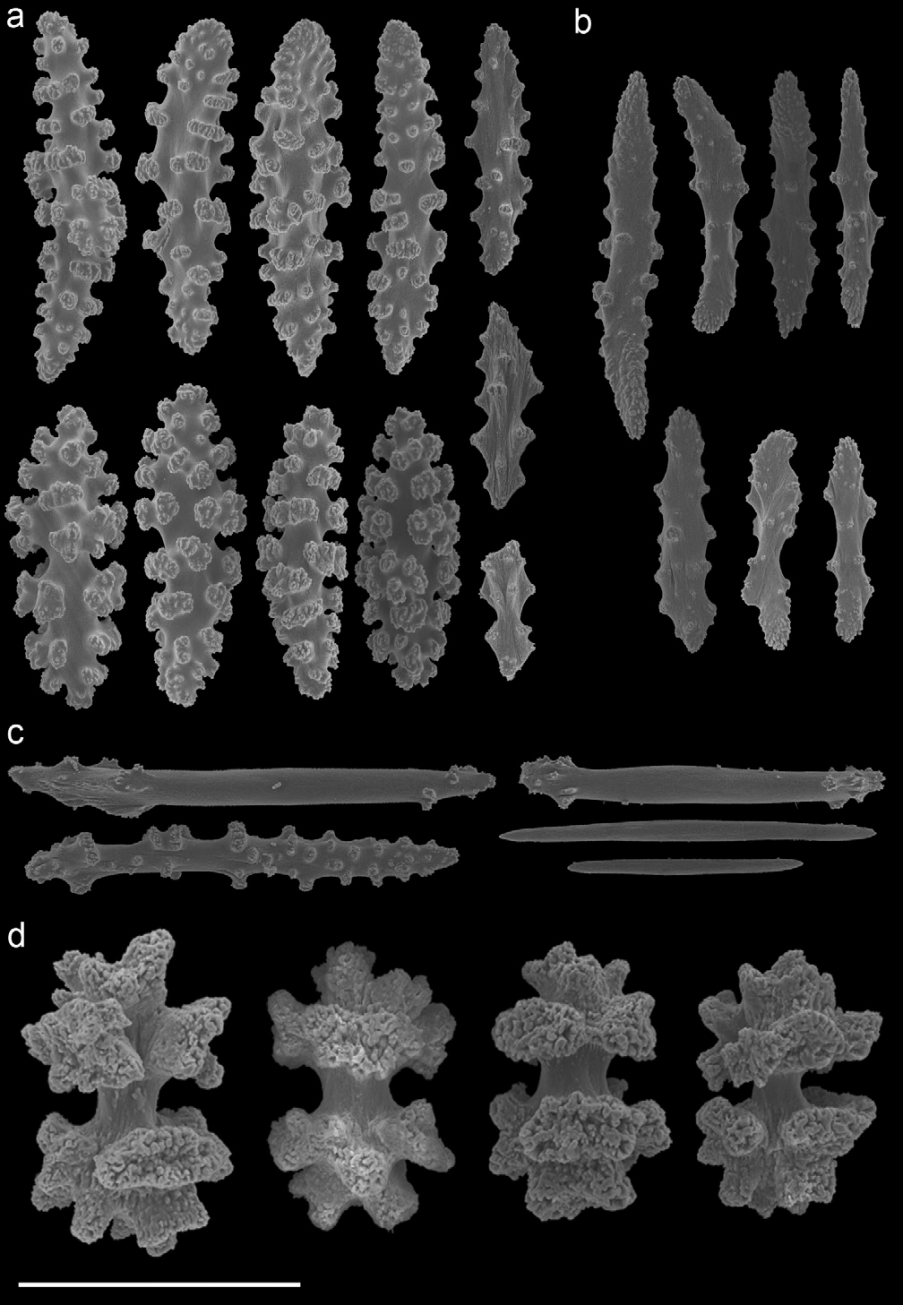
*Annella mollis* (RMNH Coel. 39875) **A** spindles from the coenenchyme **B** tentacle rods **C** medulla spindles from the axis **D** double heads from the surface layer. Scale bar represents 0.1 mm, except for **D** which is 0.05 mm.

**Figure 12.**
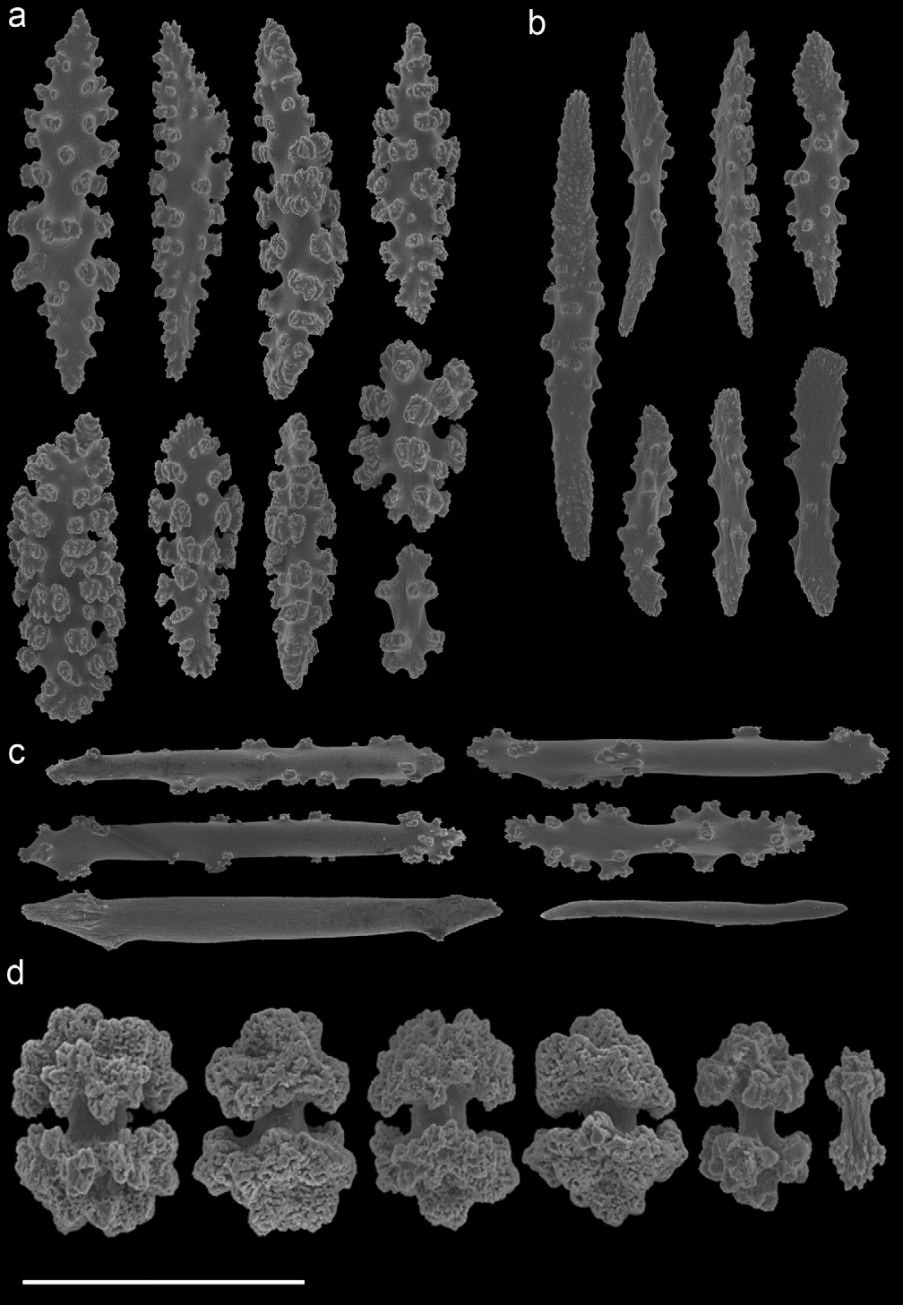
*Annella* cf. *mollis* (RMNH Coel. 39877) **A** spindles from the coenenchyme **B** tentacle rods **C** medulla spindles from the axis **D** double heads from the surface layer. Scale bar represents 0.1 mm, except for **D** which is 0.05 mm.

Previous identifications of the hosts of *Hippocampus bargibanti* may well be in error since they were made by non-gorgonian specialists, except for the identifications in Lourie and Randall (2003) which were done by Dr F.M. Bayer. Based on the herein presented data and re-examination of the holotypes, it seems plausible that the published coral host records contain several errors. For the genus *Annella* the literature records (see Table 1) show the same host species as found in the present study (Table 2, Figs 8–12), but previous identifications were based on the growth form (mesh shape) and not on the sclerites. These identifications should be re-assessed based on sclerite morphology.

Lourie and Kuiter (2008) mention *Acanthogorgia* spp. as hosts for *Hippocampus denise*, based on a photograph (Lourie and Randall 2003: image 10; pers. comm. Sara Lourie). This identification seems erroneous, since the polyps shown in the photograph are not characteristic for the genus *Acanthogorgia*. Although no certain identification can be made based on a photograph, the image most likely depicts a zoanthid (Dr James Reimer, pers. comm.), which would be the first indication that pygmy seahorses might be associated with zoanthids as well. Associations with *Echinogorgia* sp. and *Subergorgia* sp. cannot be confirmed based on material in the present study.

For many organisms molecular methods can be of help to identify species, but so far barcoding of Octocorallia for the COI gene has not been successful. Even when sequences are obtained, information on species level is very limited. Most research is currently limited to three genes, which are still unsatisfactory to identify species (McFadden et al. 2011). For Octocorallia the ‘traditional’ taxonomy based on morphological characters remains of primary importance. When new species of pygmy seahorses are described a photograph of the whole host colony, and a close-up of its polyps and branches should also be provided, which is normally enough to identify the host to family or possibly genus level. Preferably also tissue samples of the host should be collected for taxonomic studies.

### Conservation

The distribution ranges of the pygmy seahorses are largely situated within the Coral Triangle (Lourie 2001, Hoeksema 2007), which receives much attention with regard to coral reef conservation. The entire genus *Hippocampus* is listed in Appendix II of CITES and *Hippocampus bargibanti* and *Hippocampus denise* are listed as data deficient in the IUCN Red List (Lourie et al. 2004), whereas the other five pygmy seahorse species have not yet been assessed. One of the main threats to seahorse populations concerns habitat loss and degradation, especially for the species depending on specific host coral species. Seahorses have been increasingly used as flagship species in local and regional conservation programs to promote the protection of both the seahorses and their habitats (Scales 2010). Knowledge on the distribution of the host species can be beneficial for conservation efforts of their associated organisms.

## Conclusion

This paper shows that pygmy seahorses are associated with more gorgonian and hydrozoan hosts than previously assumed, resulting in new associations; *Hippocampus bargibanti* is associated with five species of the genus *Muricella*, *Hippocampus denise* is associated with three *Annella* species, and *Hippocampus pontohi* with four hydrozoan and one algae species. No new records are available for *Hippocampus severnsi*. The presumed association of colour morphs of *Hippocampus bargibanti* with certain *Muricella* species cannot be confirmed based on our present results. Future work on pygmy seahorses should preferably include more attention for their hosts, including taking tissue samples for identification by an octocoral taxonomist.

**Figure 13.**
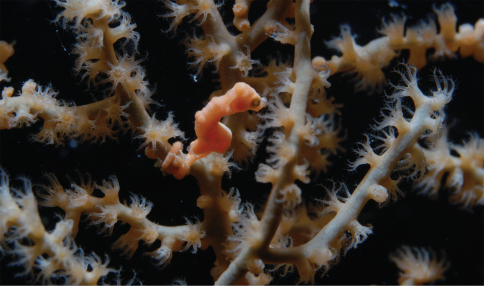
A rare occurrence, *Hippocampus denise* on *Muricella* sp. 2 (RMNH Coel. 39873) at Raja Ampat (photo F.R. Stokvis).
